# PARP-1 inhibitors enhance the chemosensitivity of leukemia cells by attenuating NF- **кB pathway activity and DNA damage response induced by Idarubicin**: PARP-1 inhibitors attenuate DNA damage response

**DOI:** 10.3724/abbs.2021011

**Published:** 2021-12-17

**Authors:** Bo Ke, Anna Li, Huan Fu, Chunfang Kong, Tingting Liu, Qingqing Zhu, Yue Zhang, Zixia Zhang, Chen Chen, Chenghao Jin

**Affiliations:** 1 Department of Hematology Jiangxi Provincial People's Hospital Nanchang 330006 China; 2 Key Biologic Laboratory of Blood Tumor Cell of Jiangxi Province Nanchang 330006 China; 3 National Clinical Research Center for Hematologic Diseases the First Affiliated Hospital of Soochow University Soochow 215006 China

**Keywords:** PARP-1 inhibitor, NF-кB pathway, leukemia, chemosensitivity, DNA damage response

## Abstract

Idarubicin (IDA), an anthracycline antineoplastic drug, is commonly used in the treatment of acute myeloid leukemia (AML) with reasonable response rates and clinical benefits. However, some patients still relapse, or do not respond, and suffer high fatality rates. Recent studies have shown that overexpression of PARP-1 may represent an important risk factor in AML patients. The aim of the present study was to determine the underlying molecular mechanisms by which the PARP-1 inhibitor Olaparib enhances the chemosensitivity of the leukemia cell line K562 and THP1 to IDA. Our data demonstrated that PARP-1 is upregulated in AML patients as well as in K562 and THP1 cells, and that the suppression of PARP-1 activity by Olaparib enhances the inhibitory effect of IDA. A mechanistic study revealed that Olaparib decreases the expressions of p-ATM, p-IκBα, XIAP and p65, and upregulates Bax, cleaved-Caspase-3 and γ-H2AX. Olaparib can enhance the induction of DNA damage by IDA, probably mediated by the inhibition of the ATM-related DNA damage response. Moreover, we also found that the nuclear translocation of p65 and the nuclear export of NEMO are inhibited when IDA and Olaparib are combined. Our results suggest that Olaparib attenuates the activity of the NF-κB pathway and decreases the DNA damage response induced by IDA. Therefore, we conclude that Olaparib is a potentially valuable chemosensitizer for leukemia patients.

## Introduction

Leukemia is a hematologic malignancy mainly resulted from the clonal proliferation of leukemia stem cells with various different cytogenetic or molecular abnormalities. Although new therapeutic strategies and targeted drugs have greatly improved the remission rate and disease-free survival of leukemia patients, there are still some patients who are insensitive to the drugs or rapidly relapse after the induction of first remission. Acute myeloid leukemia (AML) is a common tumor in adults, with the highest incidence in patients over 65 years of age and a 5-year overall survival of <10%, emphasizing the need for more effective and well-tolerated therapies for these patients [1].

Idarubicin (IDA) is a new anthracycline antineoplastic drug used for the treatment of hematologic malignancies. It has a stronger antileukemic effect and evokes less drug resistance and cardiotoxicity than daunorubicin [ 2, 3]. IDA undergoes reductive bioactivation by NADPH-cytochrome P450 reductase to generate free radicals which induce DNA strand breaks (DSBs) and exert genotoxic effects [4]. As a Topoisomerase II (Top2) inhibitor, IDA binds to Top2 and forms DNA-Top2 complex which also contributes to the inhibition of Top2 enzyme activity and the induction of DSBs [5]. Therefore, the DNA damage response plays an important role in inducing the tumor cell apoptosis caused by chemotherapeutic agents. However, some patients still suffer from relapse or chemotherapy insensitivity, which leads to low remission and high fatality rate. Recent studies have shown that enhanced DNA damage repair processes are present in cancers, responding poorly to chemotherapeutic agents. Thus, targeting these components of the DNA damage repair machinery could represent an effective treatment strategy for increasing the sensitivity to these agents.

Poly(ADP-ribose) polymerase-1 (PARP-1) is an enzyme that catalyzes the attachment of poly(ADP-ribose) (PAR) chains to glutamic acid residues on target proteins. It serves as a DNA damage-sensor by binding to DNA single-strand breaks (SSBs) and is involved in the DNA damage repair process, thus contributing to the maintenance of genomic integrity [ 6, 7]. PARP-1 participates in the regulation of DNA damage-induced NF-κB-dependent survival pathways by auto-modification and recruiting protein inhibitor of activated STATy (PIASy). The latter is required for NEMO SUMOylation and NF-κB activation in response to DNA damage [8]. Thus, PARP-1 activates NF-κB-regulated resistance to apoptosis and acts as a survival factor during DNA damage repair [9]. Olaparib, a PARP-1 inhibitor, is approved by the US Food and Drug Administration (FDA) for the treatment of ovarian and breast cancer with specific indications [ 10, 11]. Thus far, the antileukemia effects of Olaparib combined with IDA remain uninvestigated.

In the present study, K562 and THP1 leukemia cells were pre-treated with Olaparib, followed by IDA treatment. The effects of Olaparib combined with IDA on the activity of the NF-κB pathway and DNA damage response were analyzed. The results demonstrated that the activity of the NF-κB pathway is reduced by combination treatment, as evidenced by decreased expression of XIAP, a target gene of the NF-κB/p65 transcription factor, and decreased levels of phosphorylation of IκBα and the p65 subunit. Ataxia telangiectasia mutated (ATM), another sensor of DNA damage, is also inhibited by Olaparib despite otherwise being activated by treatment with IDA.

## Materials and Methods

### Cell culture

Human Leukemia cell lines (Jurkat, K562 and THP1) and lymphoma cell line (OCI-Ly19) were obtained from the Cell Bank of the Chinese Academy of Sciences (Shanghai, China), and cultured in RPMI-1640 medium containing 10% fetal bovine serum (Gibco, Carlsbad, USA) at 37°C in an incubator (SANYO, Osaka, Japan) with 5% CO _2_. IDA (Pfizer, New York, USA) was dissolved in saline as a 100 mM stock solution and stored in aliquots at –20°C. Olaparib was provided by Beyotime (Shanghai, China).

### Real-time PCR assay

Our study was approved by the Ethics Committee of Jiangxi Provincial People′s Hospital. Twenty patients diagnosed with *de novo* AML (M3 patients were excluded) and eighteen patients with other blood diseases (non-malignant hematological tumors) were recruited in the study. All patients provided signed informed consent forms. Bone marrow samples were collected and treated with erythrocyte lysis buffer. Total RNA was extracted using TRNzol Universal Reagent (TIANGEN, Beijing, China), and FastQuant RT kit (TIANGEN) was used to synthesize cDNA. Real-time PCR was performed using FastFire qPCR PreMix (TIANGEN) and gene-specific primers on a TL988 Real-Time PCR system (TIANLONG, Xi’an, China). The primers were designed using Primer 3 software and sequences are as follows: *β-actin* forward: 5′-GCATCCTCACCCTGAAGTA-3′, reverse: 5′-GAGGTAGTCAGTCAGGTCC-3′; *PARP1* forward: 5′-GCCTTCAGGAGTTGTTCTTAGC-3′, reverse: 5′-TGATACCTTCCTCCTTGACCT-3′. The relative expression of the target gene was calculated by the method of 2 ^–∆∆Ct^, with *β-actin* used as the internal reference gene.

### Cell viability assay

K562 and THP1 cells were seeded into 96-well plates at a density of 1×10 ^4^ cells/well. Cells were cultured with different concentrations of IDA in the absence or presence of Olaparib (2 μM) for 24 h. Subsequently, cell viability was measured using a cell counting kit-8 (CCK-8; AbMole, Shanghai, China). Briefly, K562 and THP1 cells were incubated with 10 μL CCK-8 reagent and then further incubated at 37°C in 5% CO _2_ for 2 h. Optical density was measured at 450 nm using a microplate reader (Rayto, Shenzhen, China).

### Cell apoptosis assay

K562 and THP1 cells were seeded into 6-well plates at a density of 2×10 ^6^ cells/well, pretreated with or without Olaparib (2 μM) for 24 h, and then further treated with IDA (50 nM) for 12 h. Subsequently, the cells were collected and washed with 1×PBS and resuspended in 100 μL 1× binding buffer (BD Biosciences, San Diego, USA). The cells were then mixed with 5 μL Annexin V-PE and 10 μL 7-AAD (BD Biosciences), followed by incubation in the dark for 15 min. Finally, the cells were assessed by flow cytometry on a Beckman flow cytometer (Beckman Coulter, Pasadena, USA).

### Analysis of apoptosis by Hoechst 33258 staining

K562 and THP1 cells were seeded into 6-well plates at a density of 2×10 ^6^ cells/well and pretreated with 2 μM Olaparib for 24 h, followed by exposure to IDA for 12 h. Cells were then washed with Buffer A (KeyGEN, Nanjing, China), fixed with 4% paraformaldehyde, and finally incubated with Hoechst 33258 (KeyGEN) according to the manufacturer’s instructions. The cells were observed and photographed under a fluorescence microscope (Leica Biosystems, Wetzlar, Germany). Apoptotic cells were identified by their abnormal fragmented or intensely stained nuclei. The apoptosis rate was defined as the ratio of positive cells to total cells.

### Immunofluorescence assay

K562 and THP1 cells were seeded into 6-well plates at a density of 2×10 ^6^ cells/well and pretreated with 2 μM Olaparib for 24 h, followed by exposure to IDA for 12 h. Cells were collected and washed with 1×PBS, and then fixed with 4% paraformaldehyde for 15 min at room temperature. The fixed cells were washed twice with 1× PBS and resuspended. A drop of the cell suspension was placed on a polylysine-coated glass slide and left to dry naturally. Then, cells were permeabilized in PBS containing 0.5% Triton X-100 for 20 min and blocked with 5% BSA for 30 min at room temperature. Cells were then incubated with antibodies specific for γ-H2AX and ATM (1:500 dilution; Abcam, Cambridge, UK) diluted in antibody dilution buffer (Boster, Wuhan, China) at 4°C overnight. Subsequently, the cells were washed with 1×PBS and incubated with Cy3-conjugated IgG secondary antibodies (1:300 dilution; ABclonal, Wuhan, China) for 1 h in the dark. Finally, the cells were washed and stained with DAPI for 5 min, and then observed and photographed under the fluorescence microscope. Fluorescence intensity was quantified by Image J software.

### Western blot analysis

Cells were washed and lysed using cell lysis buffer RIPA (Beyotime) supplemented with 1 mM Protease Inhibitor Cocktail (AbMole) and 1 mM Phosphatase Inhibitor Cocktail (APExBIO, Houston, USA). Cell lysates were subject to SDS/polyacrylamide gel electrophoresis and transferred to a PVDF membrane (GE Healthcare Life sciences, Braunschweig, Germany) using a semi-dry transfer apparatus (Bio-Rad, Hercules, USA). The membrane was blocked with 5% bovine serum albumin (BSA) for 1 h at room temperature, and subsequently incubated with specific antibodies against p-ATM, p-IκBα, XIAP, NEMO, H2AX, p65 or PARP-1 (Abcam) diluted in antibody dilution buffer at 4°C overnight. PVDF membranes were washed with PBS supplemented with 0.05% Tween-20 (PBST) and incubated with HRP-conjugated secondary antibodies (1:5000; Beyotime) for 1 h at room temperature. Finally, signals were visualized using Chemiluminescence Imaging System ChemiScope6000 (Clinx, Shanghai, China). GAPDH was used as a loading control. Intensity was quantified by Image J software.

### Comet assay

The DNA damage in K562 and THP1 cells induced by treatment with IDA was assessed by alkaline comet assay. K562 and THP1 cells were treated with Olaparib or IDA for 24 h, collected and washed with 1× PBS. Cells were resuspended in 0.7% low melting point agarose (LMA) and transferred to glass slides precoated with 0.5% normal melting point agarose (NMA). For DNA release, slides were incubated in cold alkaline lysis buffer containing 10% dimethyl sulfoxide (KeyGEN) at 4°C for 2 h. DNA unwinding took place in the alkaline electrophoretic buffer (1 mM EDTA and 300 mM NaOH) for 1 h, and then electrophoresis was performed at 25 V for 20 min. Slides were washed three times with neutralization buffer (0.4 mM Tris, pH 7.5). Finally, the slides were stained with 20 μL ethidium bromide and photographed under the fluorescence microscope. At least 50 cells were measured per treatment using CASP software ( https://casplab.com), and the damage was expressed as tail intensity (Tail DNA%) which represents the percentage of DNA in the comet tail relative to the total amount of DNA.

### Statistical analysis

Data are expressed as the mean±standard deviation (SD) and are representative of triplicate samples. Statistics were calculated using Graphpad Prism 7 software. Normally distributed groups were compared by unpaired *t*-test and one-way ANOVA. *P*<0.05 was considered statistically significant.

## Results

### PARP-1 is upregulated in AML and K562 cells, and the cytotoxicity of IDA for K562 cells is enhanced by the PARP-1 inhibitor Olaparib

**Figure 1:**
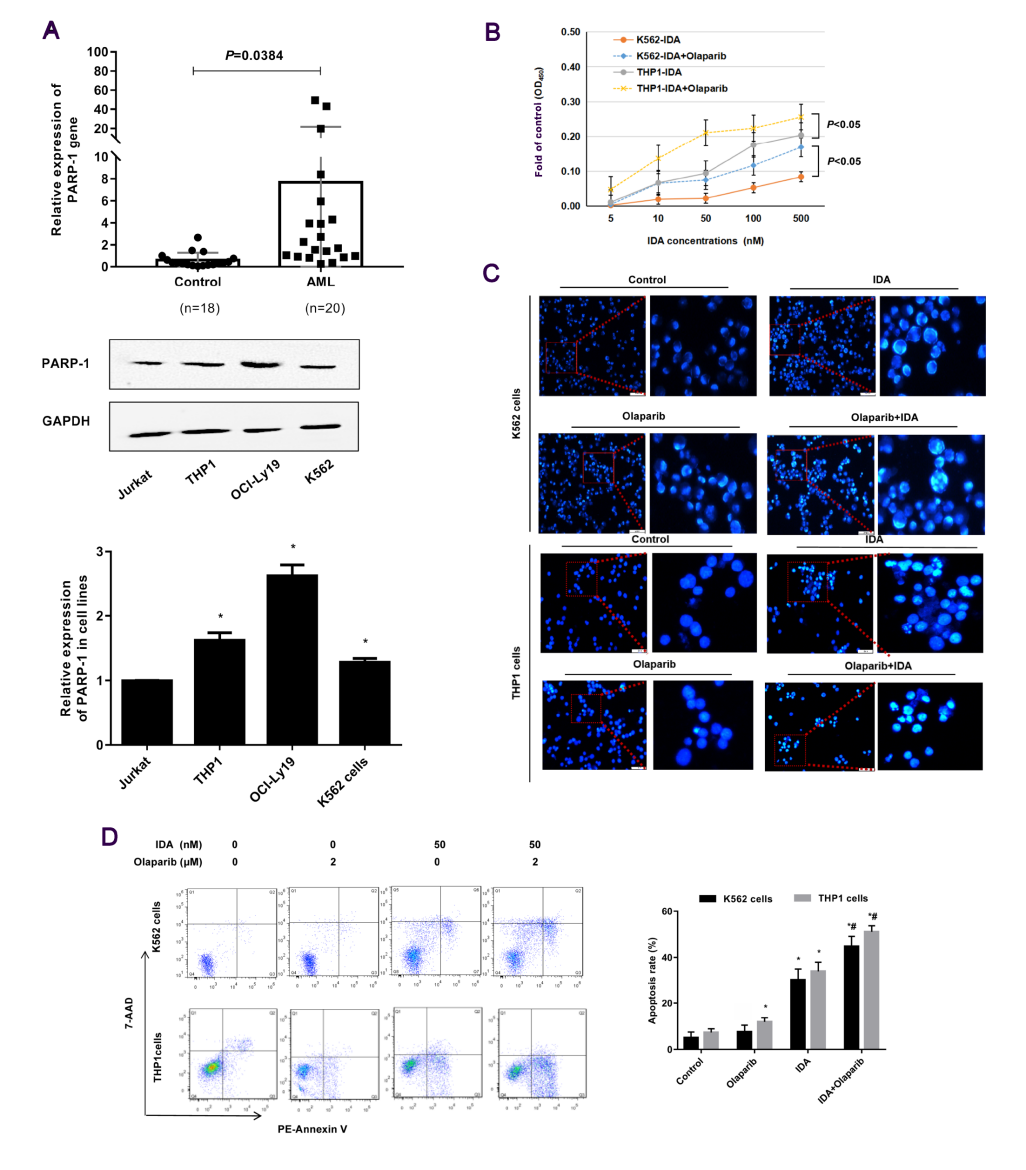
Figure1 **The PARP-1 inhibitor, Olaparib enhances the cytotoxicity of IDA in K562 and THP1 cells**(A) Expression level of the PARP-1 gene in cell lines and AML patients' cells were measured by western blot analysis and real-time PCR. (B) K562 and THP1 cells were treated with 2 μM Olaparib for 24 h, followed by exposure to IDA for 12 h. Cytotoxicity was determined by CCK-8 assay. (C) Cells were treated and harvested for Hoechst 33258 staining. Apoptotic cells are characterized by their abnormal fragmented or intensely stained nuclei. Scale bar: 100 μm. (D) Cells were treated with the indicated concentrations of Olaparib and IDA and then harvested for Annexin V-PE/7-AAD staining. Apoptotic K562 and THP1 cells were assessed by flow cytometry. Data are presented as the mean±SD from three independent experiments. *P<0.05 compared with the control group; #P<0.05 compared with the IDA group.

Western blot analysis results indicate that the expression levels of PARP-1 in the cell lines K562, THP1 and OCI-Ly19 were significantly higher than that in the Jurkat cell line ( Figure 1A). Furthermore, this high activity of PARP-1 was not limited to cell lines because AML patients′ cells also expressed high level PARP-1. This was not the case for cells from patients with other blood diseases (non-malignant hematological tumor) ( Figure 1A).

To determine the cytotoxic effect of Olaparib and IDA on K562 and THP1 cells, CCK-8 assays were performed to evaluate K562 and THP1 cell viability. Cells were pre-treated with 2 μM Olaparib for 24 h, and then exposed to different dose of IDA (5‒500 nM) for 12 h. The results showed that the viability of K562 and THP1 cells was significantly inhibited by IDA in a dose-dependent manner. As expected, treatment combining both IDA and Olaparib enhanced cytotoxicity ( Figure 1B). We next assessed the effect of Olaparib treatment together with IDA on apoptosis of K562 and THP1 cells, by performing Hoechst 33258 and PE-Annexin V/7-AAD staining, fluorescence microscopy and flow cytometry. Hoechst 33258 staining showed that the numbers of apoptotic cells were significantly increased in cultures exposed to both agents, compared with those exposed to either IDA or Olaparib alone, characterized by fragmented or intensely stained nuclei ( Figure 1C). Moreover, the proportions of 7-AAD positive cells in the population treated with both agents combined were higher than IDA treatment alone. In contrast, K562 cells treated with Olaparib did not show obvious differences in the rates of apoptosis relative to the control, as shown in Figure 1D.

### Activity of the NF-κB pathway is attenuated after treatment with IDA and Olaparib

**Figure 2:**
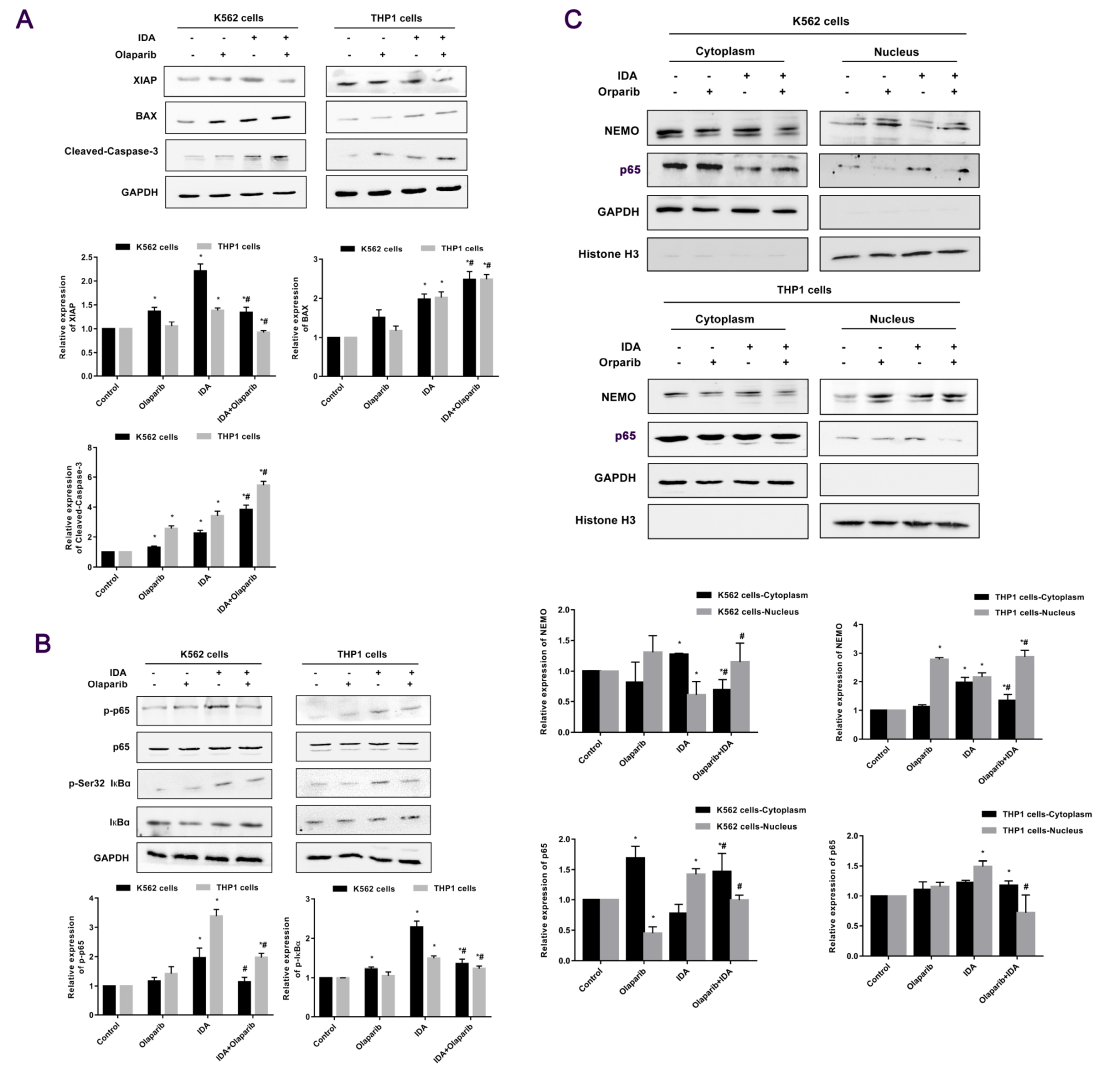
Figure2 **The activity of the NF-κB pathway is decreased after treatment with IDA and Olaparib**(A) Cells were treated and harvested, and the expression levels of downstream members of the NF-κB signaling pathway (XIAP, BAX and Cleaved-Caspase-3) were analyzed by western blot analysis. (B) Expression and phosphorylation of p65 and IкBα was analyzed by western blot analysis. (C) Nuclear translocation of p65 and nuclear export of NEMO were analyzed by western blot analysis. Data are presented as the mean±SD from three independent experiments. *P<0.05 compared with the control group; #P<0.05 compared with the IDA group.

To investigate whether the activity of the NF-κB pathway is affected by Olaparib, anti-apoptotic protein XIAP, downstream of the NF-κB pathway, was analyzed by western blot analysis. The results showed that the expression level of XIAP was increased in K562 and THP1 cells treated with IDA alone compared with control, but when the cells are exposed to both IDA and Olaparib, the expression of XIAP was decreased compared with the contril group. As a member of the BCL2 protein family, BAX forms a heterodimer with BCL2, and functions as an apoptosis activator. Accordingly, the expression of BAX was upregulated after exposure to IDA, and even more when IDA was combined with Olaparib. Additionally, caspase-3 was more highly activated after combined treatment than IDA treatment, as determined by an antibody directed against cleaved-caspase-3 ( Figure 2A). To further confirm the effect of Olaparib on the NF-κB pathway, the degree of phosphorylation of IκBα, a repressor of NF-κB activity, was measured. It was highly phosphorylated at Ser32 after IDA treatment, even more after combined treatment. Consequently, the phosphorylation level of p65 at Ser536 was also increased compared with cells treated with both agents (as shown in Figure 2B). These results also suggest that Olaparib may inhibit the NF-κB signal pathway by blocking proteasomal degradation of IκBα mediated by its phosphorylation. NEMO/IKKγ serves as a regulatory subunit of the IκB kinase (IKK) complex, required for the activation of the NF-κB pathway and involved in regulation by phosphorylation of the inhibitory subunit IκBα. Therefore, we also investigated whether the nuclear export of NEMO and the nuclear translocation of p65 caused by IDA-induced DSB are inhibited by Olaparib. Western blot analysis results showed that the nuclear translocation of p65 as well as the nuclear export of NEMO was inhibited after treatment with IDA and Olaparib together ( Figure 2C).

### Effects of IDA and Olaparib on the formation of ATM and H2AX foci

**Figure 3:**
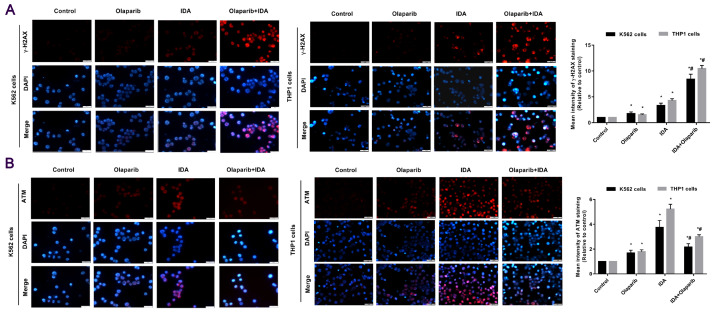
Figure3 **Olaparib increases the formation of γ-H2AX foci induced by IDA treatment, but decreases ATM foci**(A,B) Cells were harvested and fixed for immunofluorescence analysis with the indicated antibodies. Representative images showed the ATM and γ-H2AX foci induced by IDA treatment in the absence or presence of Olaparib. Mean intensities were quantified by Image J software. Scale bar: 200 μm. Red, γ-H2AX/ATM; Blue, DNA stained with DAPI. Data are presented as the mean±SD from three independent experiments. *P<0.05 compared with the control group; #P<0.05 compared with the IDA group.

Next, we addressed whether pretreatment with Olaparib has any effect on the DNA damage response in K562 and THP1 cells exposed to IDA. Immunofluorescence results showed that IDA induced the formation of γ-H2AX foci which serves as a marker of DNA DSB damage. Moreover, the formation of γ-H2AX foci was increased when K562 and THP1 cells were treated with IDA combined with Olaparib ( Figure 3A). ATM protein is mainly located in the nucleus and monitors the integrity of DNA by acting as a sensor of DNA damage. Hence, we also explored the formation of ATM foci in K562 and THP1 cells treated with Olaparib or IDA. The results showed that IDA treatment significantly increased the formation of ATM foci, but the combination of IDA and Olaparib decreased the formation of ATM foci ( Figure 3B). These results suggest that Olaparib can enhance the induction of DSBs by IDA, probably mediated by the inhibition of the ATM-related DNA damage response.

### Olaparib augments IDA-induced DNA damage but decreases phosphorylation of ATM

**Figure 4:**
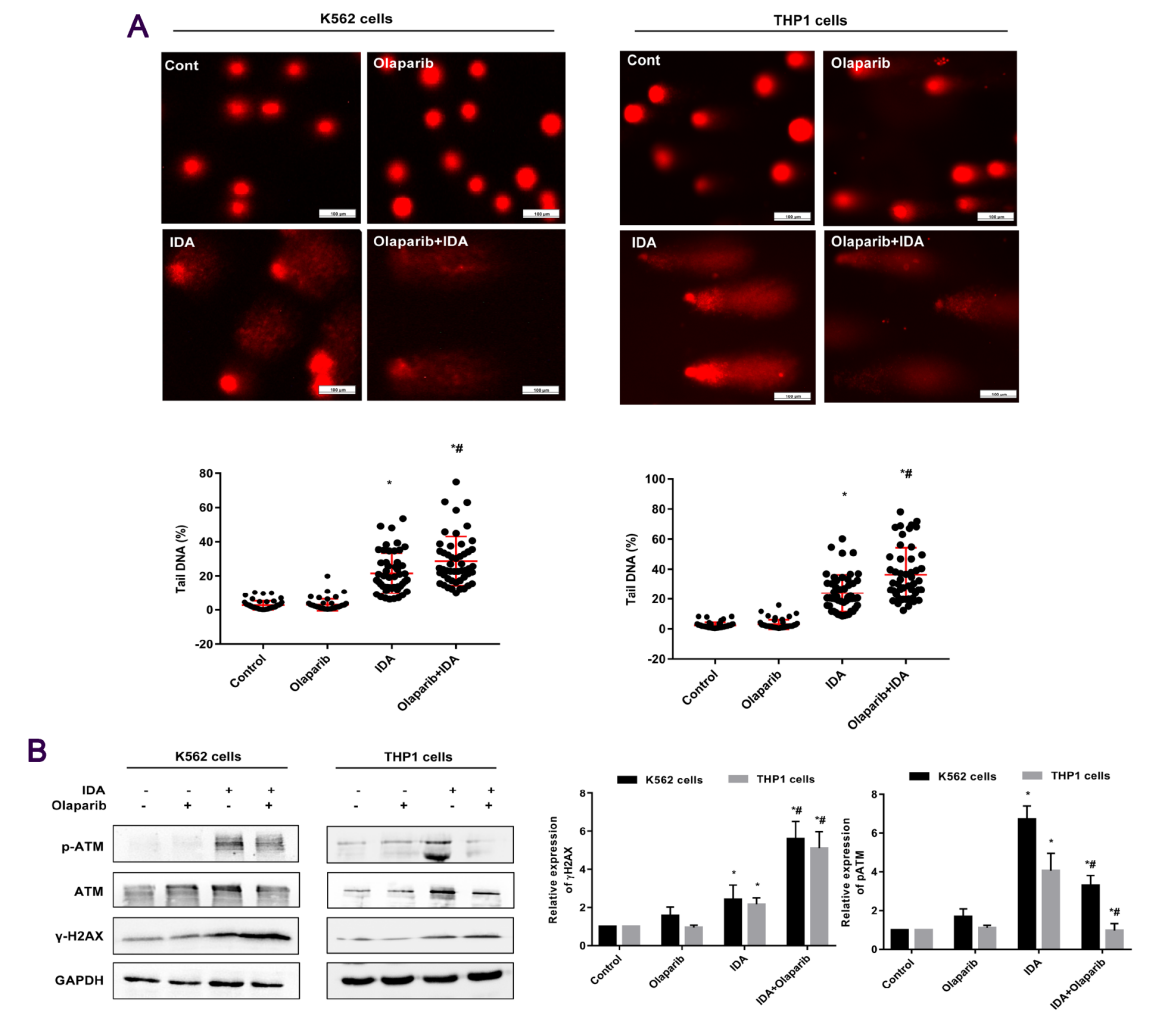
Figure4 **Effects of Olaparib on the IDA-induced DNA damage response in K562 and THP1 cells**(A) Cells were treated and harvested for the Comet assay after exposure to IDA. A total of 50 cells was measured per treatment using CASP software, and the damage was expressed as tail intensity (Tail DNA%). Scale bar: 100 μm. *P<0.05. (B) Expression and phosphorylation of ATM and γ-H2AX were analyzed by western blot analysis. Data are presented as the mean±SD from three independent experiments. *P<0.05 compared with the control group. #P<0.05 compared with the IDA group.

Further work aimed at investigating the impact of Olaparib combined with IDA on DNA damage, which is assessed by the alkaline comet assay. Mock- and Olaparib-treated cells did not show any differences in comet tail intensity (Tail DNA%), which was 2.7% and 3.0% in K562 cells, 2.3% and 3.0% in THP1 cells, respectively. However, a significant increase in tail intensity was observed in cells treated with IDA (22% and 24%) compared with the control, which was further increased by exposure to both Olaparib and IDA (29% and 36%) in K562 and THP1 cells, respectively ( Figure 4A).

To investigate the molecular mechanisms involved in IDA-induced DNA damage, the expression of proteins such as p-ATM and γ-H2AX involved in the DNA damage response was assayed by western blot analysis. The results showed that the phosphorylation of ATM was increased in K562 and THP1 cells treated with IDA compared with control group, but the phosphorylation of ATM was decreased when IDA was combined with Olaparib compared with IDA treatment ( Figure 4B). However, the degree of phosphorylation of H2AX was increased when IDA and Olaparib were combined. These data are consistent with the results from the immunofluorescence assays. The results also strongly suggest that Olaparib may have the effect of inhibiting DNA damage repair probably by blocking the activity of ATM.

## Discussion

PARP-1, a specific sensor protein, detects DNA lesions and regulates complex networks of cellular processes in response to DNA damage. PARP-1 is rapidly activated and recruited to the damaged sites in response to DNA damage, which is a critical early event for DNA damage repair [12]. Olaparib upregulates the expression of death receptor TRAIL-R2 and significantly increases tumor cell sensitivity to NK killing and antibody-dependent cellular cytotoxicity (ADCC) in both BRCA WT and mutant prostate carcinoma cells [13]. Recent studies have shown that overexpression of PARP-1 may represent an important risk factor and predict poor overall survival (OS) and relapse-free survival (RFS) in AML patients [ 14, 15]. In the present study, PARP-1 was also found to be highly expressed in tumor cells from AML patients and in K562 cells. We then used the PARP-1 inhibitor Olaparib to treat leukemia K562 cells *in vitro*, either alone or combined with IDA. The results showed that the combination of drugs led to significant apoptosis and increased growth inhibition. In view of the pivotal role of PARP-1 in DNA repair, we hypothesized that PARP-1 inhibition might influence sensitivity to chemotherapy, particularly to DNA-damaging agents. Furthermore, our study revealed that the activity of the NF-κB pathway was attenuated when cells were treated with IDA together with Olaparib. This may indicate that PARP-1 plays a more critical role in the process of DNA damage-induced NF-κB signal pathway activation, probably acting by inhibiting the activation and nuclear export of NEMO.

ATM is a nuclear protein kinase that also plays a crucial role in the DNA damage response after DNA DSBs. In response to DSBs, ATM is activated and recruited to the site of the damage, subsequently phosphorylating a series of substrates, including H2AX, Chk2, and p53. These phosphorylated substrates promote cell cycle arrest and initiate DNA repair to prevent genomic instability or induce apoptosis when the damage is beyond repair [16]. PD-L1 upregulation in cancer cells has been observed, which is also a response to DSBs, and this process requires the activation of ATM/ATR/Chk1 kinases after IR or genotoxic treatment [17]. Genotoxic chemotherapy-induced DNA damage can elicit the activation of pro-survival signaling pathways and promote the acute release of pro-survival chemokines, contributing to the formation of chemoprotective microenvironments [18]. Thus, improved chemotherapeutic regimens require not only cytotoxic drugs, but targeted therapeutic agents that inhibit pro-survival signaling. Recent studies have shown that the ATM-dependent NF-κB pathway is activated after chemotherapy, which induces the expression of niche-protecting cytokines in both ALL cell lines and primary cells *in vitro*. Pharmacological or genetic inhibition of ATM-dependent NF-κB pathway signaling reversed the upregulation of these cytokines and sensitized ALL cells to chemotherapeutics [19].

In the present study, we showed that Olaparib impaired the activation of ATM, as evidenced by the reduced formation of ATM foci and downregulated phosphorylation induced by IDA, leading to the accumulation of unrepaired DNA damage and cell apoptosis. Activated PARP-1 initiates a cascade that triggers PIASy-dependent SUMOylation of IKKγ (NEMO), which results in nuclear-to-cytoplasmic IKKγ activation and NF-κB/p65 nuclear translocation [9]. Thus, we hypothesized that blocking the activity of PARP-1 could inhibit the IKKγ/NF-κB signaling pathway, which is dependent on the activation of ATM. Our results indicated that the attenuated activity of the NF-κB pathway may be a consequence of the greater stability of the inhibitor IκBα. The activity of the NF-κB pathway is modulated by the inhibitor IκBα, which is negatively regulated by the IKB kinase (IKK) complex consisting of two catalytic subunits, IKKα and IKKβ, and a regulatory subunit IKKγ/NEMO [ 20, 21]. NEMO associates with activated ATM after the induction of DNA DSBs. ATM phosphorylates NEMO at the Ser85 residue to promote its ubiquitin-dependent nuclear export [ 22, 23].

Nevertheless, there are still some limitations in this study. The mechanism by which the PARP-1 inhibitor regulates ATM activation has not been elucidated. Whether the attenuated activation of NF-κB signaling pathway depends on the nuclear export of NEMO has not been established. The function of Olaparib needs to be validated in other cell lines or *in vivo* animal experiments.

**Figure 5:**
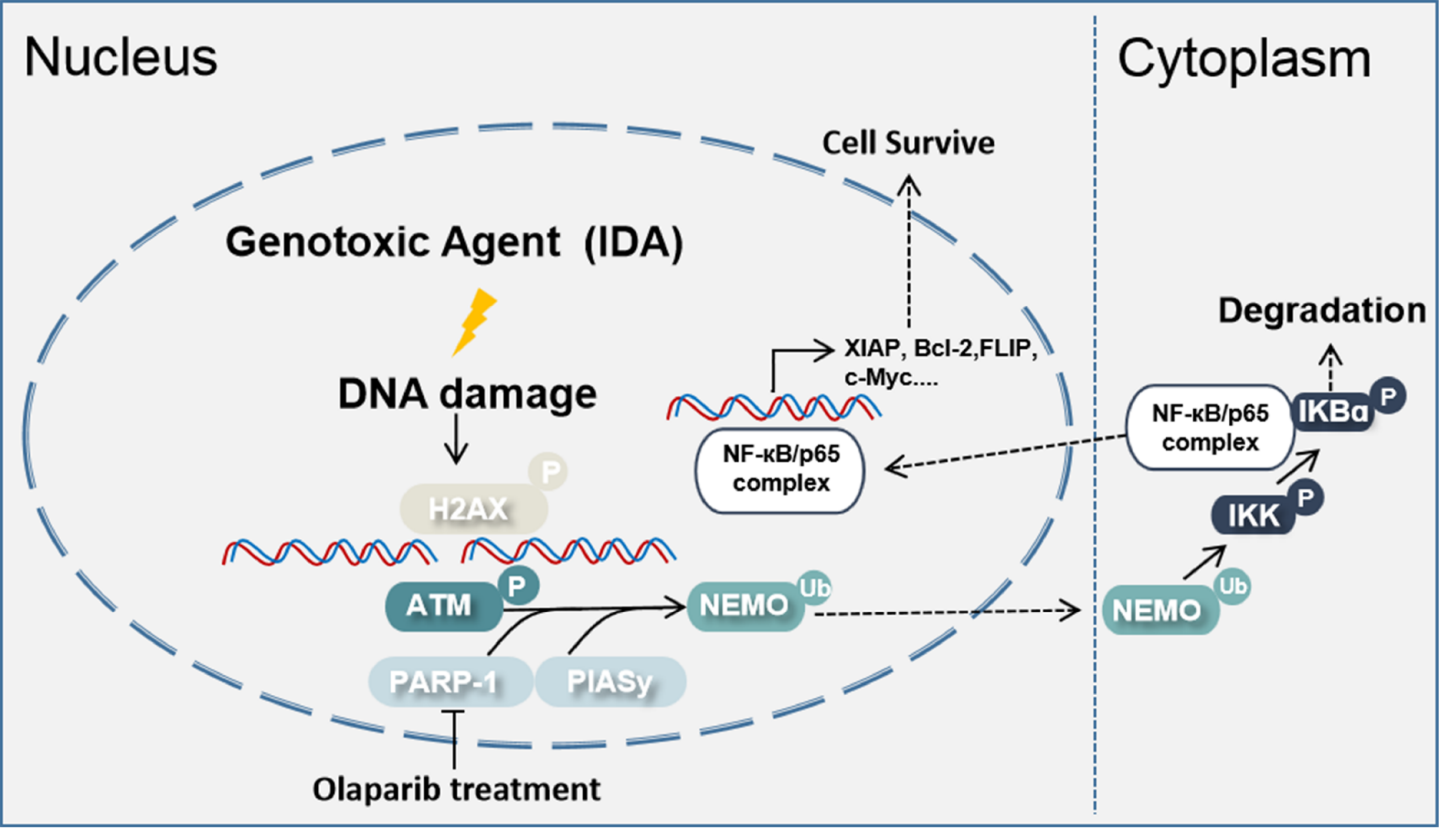
Figure5 **Schematic representation of the pathway of the enhancement of chemosensitivity in leukemia cells by Olaparib treatment**As a Topoisomerase II (Top2) inhibitor, IDA binds to Top2 and forms DNA-Top2 complex which also contributes to the inhibition of Top2 enzyme activity and the induction of DSBs. Activated PARP-1 in leukemia cells initiates a cascade that triggers PIASy-dependent SUMOylation of IKKγ (NEMO), which results in nuclear-to-cytoplasmic IKKγ activation and NF-κB/p65 nuclear translocation. Thus, PARP-1 activates NF-кB-regulated resistance to apoptosis and acts as a survival factor during DNA damage repair. Blocking the activity of PARP-1 with Olaparib inhibits the IKKγ/NF-κB signaling pathway, which is dependent on the activation of ATM. ATM phosphorylates NEMO at the Ser85 residue to promote its ubiquitin-dependent nuclear export. PARP-1 inhibitor Olaparib exerts a chemosensitization effect when combined with IDA.

In conclusion, in the present study, we demonstrated that PARP-1 is highly expressed in AML patients′ cells and K562 and THP1 cells, and that the inhibitor Olaparib exerts a chemosensitization effect when combined with IDA. Notably, we also demonstrated that the activation of ATM upon IDA-induced DNA damage is inhibited by Olaparib, accompanied by the blockade of the NF-κB signaling pathway and nuclear export of NEMO ( Figure 5). Therefore, we propose that understanding the molecular mechanisms underlying the response to DSB induction by chemotherapeutic agents, which leads to PARP-1-mediated IKKγ/NF-κB signal pathway activation, is of great significance for improving the chemotherapeutic regimens required for successfully treating leukemia patients.

## References

[1] Ferrara F, Schiffer CA (2013). Acute myeloid leukaemia in adults. Lancet.

[2] Marczak A, Kowalczyk A, Wrzesień-Kus A, Robak T, Jóźwiak Z (2006). Interaction of doxorubicin and idarubicin with red blood cells from acute myeloid leukaemia patients. Cell Biol Int.

[3] Lotfi K, Zackrisson AL, Peterson C (2002). Comparison of idarubicin and daunorubicin regarding intracellular uptake, induction of apoptosis, and resistance. Cancer Lett.

[4] Celik H, Arinç E (2008). Bioreduction of idarubicin and formation of ROS responsible for DNA cleavage by NADPH-cytochrome P450 reductase and its potential role in the antitumor effect. J Pharm Pharm Sci.

[5] Bigioni M, Zunino F, Capranico G (1994). Base mutation analysis of topoisomerase II-idarubicin-DNA ternary complex formation. Evidence for enzyme subunit cooperativity in DNA cleavage. Nucl Acids Res.

[6] Hassa PO, Hottiger MO (2008). The diverse biological roles of mammalian PARPS, a small but powerful family of poly-ADP-ribose polymerases. Front Biosci.

[7] Fisher AEO, Hochegger H, Takeda S, Caldecott KW (2007). Poly(ADP-ribose) polymerase 1 accelerates single-strand break repair in concert with poly(ADP-ribose) glycohydrolase. Mol Cell Biol.

[8] McCool K, Miyamoto S (2009). A PAR-SUMOnious mechanism of NEMO activation. Mol Cell.

[9] Stilmann M, Hinz M, Arslan SC, Zimmer A, Schreiber V, Scheidereit C (2009). A nuclear poly(ADP-ribose)-dependent signalosome confers DNA damage-induced iκB kinase activation. Mol Cell.

[10] Kim G, Ison G, McKee AE, Zhang H, Tang S, Gwise T, Sridhara R (2015). FDA approval summary: olaparib monotherapy in patients with deleterious germline
*BRCA*-Mutated advanced ovarian cancer treated with three or more lines of chemotherapy. Clin Cancer Res.

[11] Zimmer AS, Gillard M, Lipkowitz S, Lee JM (2018). Update on PARP inhibitors in breast cancer. Curr Treat Options Oncol.

[12] Caron MC, Sharma AK, O′Sullivan J, Myler LR, Ferreira MT, Rodrigue A, Coulombe Y (2019). Poly(ADP-ribose) polymerase-1 antagonizes DNA resection at double-strand breaks. Nat Commun.

[13] Fenerty KE, Padget M, Wolfson B, Gameiro SR, Su Z, Lee JH, Rabizadeh S (2018). Immunotherapy utilizing the combination of natural killer– and antibody dependent cellular cytotoxicity (ADCC)–mediating agents with poly (ADP-ribose) polymerase (PARP) inhibition. J Immunother Cancer.

[14] Li X, Li C, Jin J, Wang J, Huang J, Ma Z, Huang X (2018). High PARP-1 expression predicts poor survival in acute myeloid leukemia and PARP-1 inhibitor and SAHA-bendamustine hybrid inhibitor combination treatment synergistically enhances anti-tumor effects. EBioMedicine.

[15] Pashaiefar H, Yaghmaie M, Tavakkoly-Bazzaz J, Ghaffari SH, Alimoghaddam K, Momeny M, Izadi P (2018). *PARP-1* Overexpression as an Independent Prognostic Factor in Adult Non-M3 Acute Myeloid Leukemia. Genet Testing Mol Biomarkers.

[16] Summers KC, Shen F, Sierra Potchanant EA, Phipps EA, Hickey RJ, Malkas LH. Phosphorylation: the molecular switch of double-strand break repair.
*Int J Proteomics* 2011, 2011: 373816.

[17] Sato H, Niimi A, Yasuhara T, Permata TBM, Hagiwara Y, Isono M, Nuryadi E (2017). DNA double-strand break repair pathway regulates PD-L1 expression in cancer cells. Nat Commun.

[18] Gilbert LA, Hemann MT (2010). DNA damage-mediated induction of a chemoresistant niche. Cell.

[19] Chen YL, Tang C, Zhang MY, Huang WL, Xu Y, Sun HY, Yang F (2019). Blocking ATM-dependent NF-κB pathway overcomes niche protection and improves chemotherapy response in acute lymphoblastic leukemia. Leukemia.

[20] Grivennikov SI, Greten FR, Karin M (2010). Immunity, inflammation, and cancer. Cell.

[21] Medunjanin S, Putzier M, Nöthen T, Weinert S, Kähne T, Luani B, Zuschratter W (2020). DNA-PK: gatekeeper for IKKγ/NEMO nucleocytoplasmic shuttling in genotoxic stress-induced NF-kappaB activation. Cell Mol Life Sci.

[22] Wu ZH, Shi Y, Tibbetts RS, Miyamoto S (2006). Molecular linkage between the kinase ATM and NF-κB signaling in response to genotoxic stimuli. Science.

[23] Wu G, Song L, Zhu J, Hu Y, Cao L, Tan Z, Zhang S (2018). An ATM/TRIM37/NEMO axis counteracts genotoxicity by activating nuclear-to-cytoplasmic NF-κB signaling. Cancer Res.

